# Quality of Family Planning Counseling in Ethiopia: Trends and determinants of information received by female modern contraceptive users, evidence from national survey data, (2014- 2018)

**DOI:** 10.1371/journal.pone.0228714

**Published:** 2020-02-10

**Authors:** Gili Hrusa, Mark Spigt, Tariku Dejene, Solomon Shiferaw

**Affiliations:** 1 Department of Family Medicine, Faculty of Health, Medicine and Life Sciences, Maastricht University, Maastricht, The Netherlands; 2 Department of Community Medicine, General Practice Research Unit, the Arctic University of Norway, Tromsø, Norway; 3 Center for Population Studies, College of Development Studies, Addis Ababa University, Addis Ababa, Ethiopia; 4 Department of Reproductive Health and Health Service Management, School of Public Health, College of Health Sciences, Addis Ababa University, Addis Ababa, Ethiopia; Jinan University, CHINA

## Abstract

**Background:**

Family planning counseling is critical for women to make informed reproductive and sexual health decisions. Despite Ethiopia's success in expanding access to family planning services, information on the quality of family planning counseling is limited. The objectives of this study were to assess whether the quality of counseling from the female client´s perspective has changed over time (2014 to 2018) and to investigate determinants associated with the quality of counseling to provide a more nuanced understanding of disparities in sexual and reproductive health outcomes in Ethiopia.

**Methods:**

Data were obtained from five rounds of the Ethiopian Performance Monitoring and Accountability 2020 female survey questionnaire. Quality of counseling was categorized into four levels based on the responses of the questions that compose the Method Information Index, a core Family Planning 2020 indicator that serves as a proxy for quality of counseling and reflects the extent to which women are informed about side effects and alternate methods. The proportion of female contraceptive users that received good counseling were examined over time by each region, demographic characteristics, and contraception method type and source. Ordinal logistic regression was applied to the last survey round (2018) to investigate determinants associated with counseling quality.

**Results:**

The proportion of female contraception users that reported receiving information on all three questions did not significantly change over the period 2014 to 2018. Overall quality of counseling on family planning was low, with only 30% of women reporting receiving sufficient information during counseling. The likelihood of good quality counseling was the least among those who had no formal schooling when compared to those who had higher educational attainment (OR = 0.70, 95% CI: 0.50, 0.97). Women from the wealthiest quintile were 1.72 times more likely (95% CI: 1.10, 2.69) to receive good quality counseling when compared to women in the lower wealth quintile. Women from rural areas were 1.51 times more likely to have received good counseling when compared to those in urban areas (95% CI: 1.04, 2.18). Amhara residents were less likely to receive good counseling when compared to the SNNPR (OR: 0.51, 95% CI: 0.32, 0.81). Women who acquired their method from the private sector had worse counseling (OR: 0.31, 95% CI: 0.23, 0.41) when compared to the public sector. Those using short-acting methods were more at risk of receiving lesser quality counseling when compared to users of long-acting methods (OR: 0.58, 95% CI: 0.46, 0.72).

**Conclusion:**

The results of this analysis indicated that Ethiopia’s overall progress in modern contraceptive use has not been accompanied by a corresponding increase in the quality of family planning counseling. Improving the quality of contraception counseling for women across all demographics, including wealth quintiles and education, is a crucial strategy to support positive reproductive health outcomes with a rights-based focus. Based on the findings of this study, it is essential to emphasize the need to do proper counseling for all methods including short-acting methods especially for those working the private sector and some of the regions which have lower prevalence of good counseling. Further community-based participatory and qualitative research should focus on understanding the root causes and barriers to the delivery of high-quality counseling in Ethiopia.

## Introduction

In 1994 the International Conference on Population and Development in Cairo adopted a human rights approach in declaring family planning (FP) a core part of reproductive health while emphasizing the importance of quality in FP services and care [1]. Despite the consensus reached at the conference, family planning remained in the shadow of other global health challenges [2]. It was not until the London Family Planning Summit in 2012 that a bold initiative coined Family Planning 2020 (FP2020) was put forth to foster support and commitment to FP on an international scale. The global partnership called for universal access to FP services to girls and women worldwide [3].

Barriers to increased access to family planning and contraception include and are not limited to restrictive religious and cultural norms, levels of education, access to healthcare, and poor quality of FP services [4]. Quality in FP services encompasses facility-level structural components, such as the availability of services and contraception methods, and components related to the client such as privacy during consultations, waiting times, and information received during FP counseling [5]. The role of counseling in FP is to support a woman in navigating the process of choosing a contraceptive method that will allow her to fulfill her family planning goals and exercise her reproductive health rights. Clients must be appropriately informed about the contraception methods available to them and understand their efficacy, side effects, and management. The decision for a client to use contraception should ultimately be driven by the exposure to proper information to make an informed choice free from coercion [6].

Different aspects of quality of family planning services have been a focus of studies since its determination as an important component of contraception use and/or continuation, with women who reported having experienced higher quality of care have higher rates of contraceptive use and continuation [7, 8, 9, 10, 11]. A study across 15 sub-Saharan countries found that within one year of starting a contraceptive method, 7–27% of women ceased to use contraception for reasons related to the information received and confusion about side effects [7]. The evidence from studies also indicates that the provision of information on side effects is associated with improved contraceptive use outcomes [12, 13]. It is, therefore imperative to provide high-quality counseling that fosters the provision of proper information about family planning and contraception to clients.

As a participant of FP2020, the Ethiopian government committed to provide rights-based family planning with a focus on improving the quality of services and care. Ethiopia has enjoyed success in family planning in overall terms with increases in modern contraceptive use rates and an estimated decrease in unmet need from 37% to 24% [14, 15]. Nevertheless, disparities in family planning use, access, and issues surrounding suboptimal quality in services remain as challenges across the country [16, 17].

The quality of structural components of family planning services in public facilities (i.e. number of methods available, distance to facility) has been identified as an important factor in the adoption of modern contraception use in Ethiopia [15, 18]. Nevertheless, studies have suggested that the quality and coverage of family planning counseling remains low in Ethiopia. A study set in a prenatal care clinic in Gondar, Ethiopia, showed that only 34.8% of patients were counseled on family planning [19]. Another study found that the technical quality of family planning measured by the average score of the observed recommended clinical actions conducted during FP consultations was low at the national level with all 24 recommended clinical steps provided only one-third of the time [20].

Despite studies focusing on the quality of FP services in Ethiopia, they do not capture quality from the client’s perspective on information received during counseling. Assessments of counseling concerning a client’s perspective have traditionally been done either through direct observation, exit interviews, or retrospective reports by family planning clients. Asking women about the information they received can be used as a proxy for the quality of the services provided, and nationally representative surveys make it possible to study individual response data that can, in turn, shed light on quality from the client’s perspective [21]. The Method Information Index (MII), a core indicator of the FP2020 initiative, provides insight into the quality of FP counseling and reflects the extent to which women are informed about side effects and alternate methods. It is calculated from contraceptive users’ responses to three questions pertaining to the information they were given at the time they selected their contraceptive method. The reported value or “score” is the percent of women who responded “yes” to all three questions and held the assumption that essential information on FP and contraception was provided during counseling [21, 22].

While the importance of proper information received during counseling is recognized, little research has been conducted on whether information exchange has evolved over time nor how it varies across groups in Ethiopia. Comparing Demographic Health Survey (DHS) data from 2005 and 2014, Jain (2016) did not find a significant change in the MII for Ethiopia, indicating that nationwide progress in the quality of counseling from the client’s perspective had not taken place and that variations in MII scores were present based on residence, wealth, and educational attainment [23]. To address this gap and build on Jain’s findings from DHS data, this paper assessed whether changes in the quality of counseling from a client’s perspective occurred in Ethiopia from 2014 to 2018 using the Performance Monitoring and Accountability 2020 (PMA2020) national survey. The second objective was to investigate determinants associated with the quality of counseling to provide a more nuanced understanding of disparities in sexual and reproductive health outcomes driven by quality of care in Ethiopia.

### Conceptual framework

**Fig 1:**
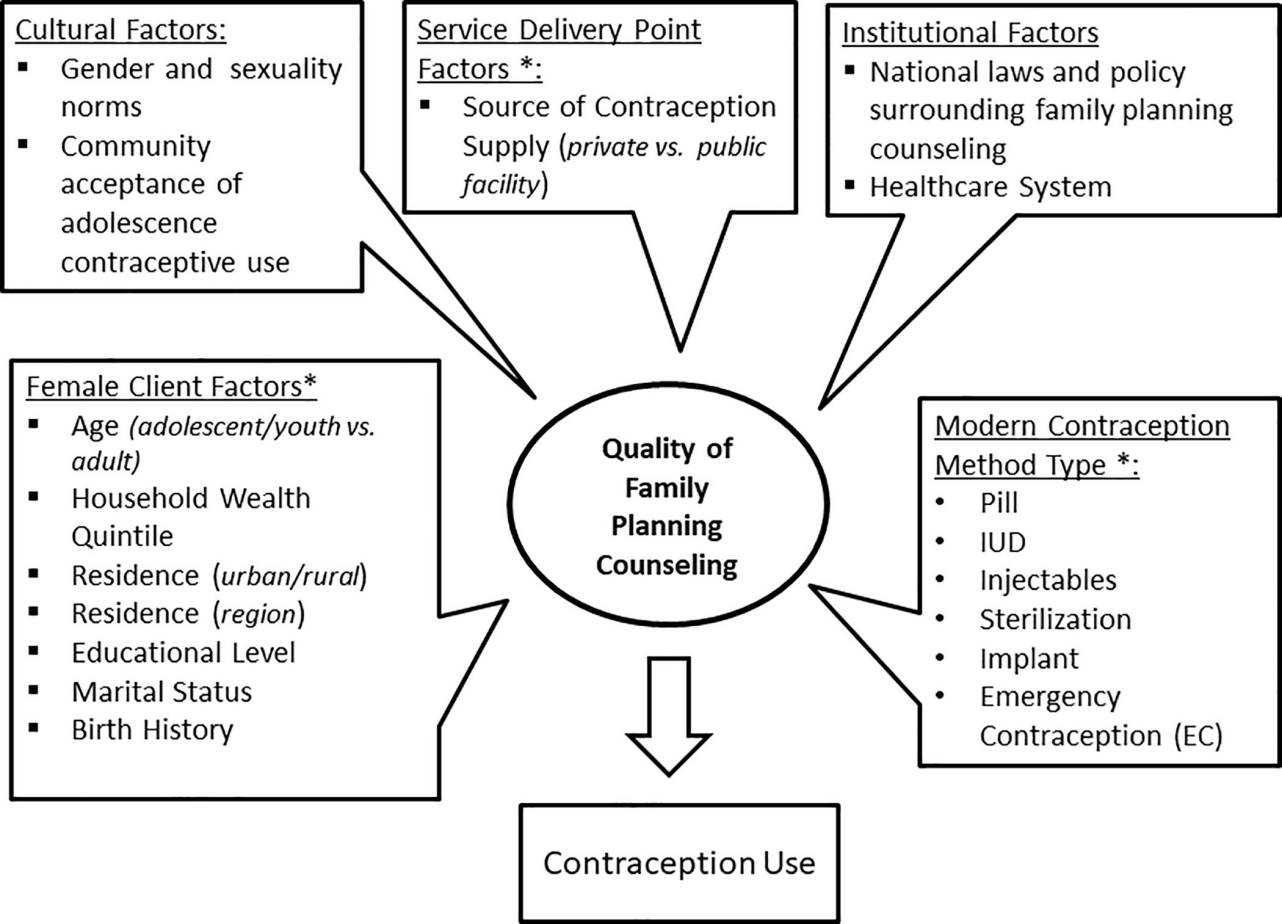
Conceptual framework.

Fig 1 diagrams the conceptual framework that grounded this analysis and depicts the relationship between quality in contraception counseling and specific attributes of the female client and other contraception method-specific factors. The elements labeled with an asterisk were directly relevant to this study. The framework was guided by Judith Bruce’s quality-of-care framework, which has endured as an inspiration behind the concept of quality in family planning specifically [24]. The Bruce framework was the first to explicitly focus on content provided by practitioners during counseling as an important element of quality in FP care and contraceptive use [25].

## Methods and materials

### Data source and sampling

Data were obtained from the last five survey rounds (2014 to 2018) of the Ethiopian Performance Monitoring and Accountability 2020 (PMA2020) female survey questionnaire. The PMA2020 survey platform was implemented in order to track progress towards FP2020 goals by multiple countries and interdisciplinary teams formed through partnerships between the Bill and Melinda Gates Institute, Johns Hopkins Bloomberg School of Public Health, and national universities and research organizations. The PMA2020 surveys were nationally representative and based on a two-stage cluster design with urban-rural and major regions as strata. The survey was based on smart phone-assisted technology to collect and transmit data to update key family planning indicators every six to twelve months in priority countries, including Ethiopia. For each enumeration area, households were selected randomly and eligible female between the ages of 15 to 49 residing in the households were administered the female household questionnaire after informed consent was obtained. The female questionnaire collected information on socio-demographic characteristics and contraceptive knowledge, use, history, and intention. Addis Ababa University’s School of Public Health was the lead implementer of the PMA2020/Ethiopia [26]. Full details of PMA2020 sampling and survey methodology has been published elsewhere [27].

### Outcome variable: Quality of counseling

The analysis was restricted to current and recent modern contraceptive users that completed the PMA2020 female questionnaire of the five rounds assessed. Female respondents were classified by the quality of counseling in four categories levels of based on the number of “yes” (coded 1) responses to the equally weighted questions that form the basis of the Method Information Index (MII). The three indicator questions were: 1) At that time (of using most recent method), were you told by the family planning provider about methods of family planning other than the ‘most recent/current method’ that you could use? 2)When you obtained your ‘most recent/current method’, were you told by the provider about side effects or problems you might have with a method to delay or avoid getting pregnant? 3) Were you told what to do if you experienced side effects or problems?

No counseling refers to no information received across all three questions. Poor counseling was defined as being informed on only one of the three indicator questions, intermediate counseling on two questions, and good counseling when the information of all three questions were addressed during counseling from the female client´s perspective. This categorization allowed us to look at the whole spectrum of quality of counseling to see the severity of the problem of counseling from no counseling to excellent counseling with all key information covered.

### Independent variables

The variables labeled with an asterisk (_*_) in the conceptual framework (Fig 1) were available in the PMA2020 data set for analysis. Age was categorized as adolescents (15 to 19 years), young adults (20 to 24 years), and adults (25 to 49 years). Household wealth quintile (lowest, low, middle, high, highest), educational status (none, primary, secondary and higher), marital status (married, not currently in union, never married), birth history (i.e., number of times given birth, 0 births, 1–2 births, 3 plus births), and residence (urban/rural) were all variables investigated. By design, the PMA2020 survey generates regional indicators for 5 major regions in Ethiopia: 1). Oromiya, 2.) Amhara, 3.) Tigray, 4.) Southern Nations, Nationalities and Peoples Region (SNNPR), and 5.) Addis Ababa. The remaining smaller regions of Gambella, Afar, Somali, Benishangul-Gumuz, and Dire Dawa were combined and categorized together as the sixth region ‘Other.’

Contraception-specific variables were method type and source. The analysis was restricted to the modern contraception methods relevant to the three MII questions due to the depth of counseling required when compared to other traditional, less invasive, and non-hormonal barrier methods such as condoms. The six included methods were: oral contraceptives, injectables, female sterilization, intrauterine devices (IUDs), emergency contraception (EC), and implants. In PMA2020 surveys, specific contraception brand names were not mentioned, and IUDs were not differentiated between hormonal and copper types. Source of method was categorized into private and public (government) sectors. The private included both for-profit and the not for profit distributors, as well as commercial outlets such as pharmacies and drugstores, which provide mainly short-acting methods such as oral contraceptives and emergency contraception in Ethiopia. Women who responded to the source of method as ‘friends/relative’ or ‘other’ (not specified) were excluded and comprised less than 1% of the contraception source categories.

### Statistical analysis and model fitting

Descriptive analysis was done for the sample across all the years with counseling quality. Trends in the proportion of contraceptive users receiving good quality counseling were examined across time (2014 to 2018), between regions, method type, source, and women’s characteristics to assess differences between groups (95% confidence intervals indicated).

To study the determinants of quality of counseling, bivariate and multivariate regression analysis for ordinal responses was applied to calculate the odds ratio (OR) and adjusted odds ratio (aOR) of quality of counseling. The generalized ordered logit model is one of the most commonly used and appropriate models for the analysis of an ordinal outcome from survey data. [28]. The model estimates the odds of being beyond or below a level of the dependent variable and the variables exert the same effect on each cumulative logit regardless of the cut off value for counseling quality. The classic ordinal proportional odds model cannot be applied if one of the predictor variables violates the proportional odds (PO) assumption. The Brant test is typically used to determine whether the assumption holds, but due to the survey data being weighted, the Wald test was more appropriate. Therefore, each variable was first tested individually to see if the requirements of the proportional odds assumption were satisfied. The year of survey and contraception method type both violated the PO assumption. Therefore, the analysis was applied to the last survey year only (2018). Method type was reclassified into two commonly used categories based on their method of action: short-acting (SA) methods (injectables, oral contraceptives, emergency contraception) and long-acting (LA) methods (IUDs, implants, and female sterilization). The reclassification of method type did not violate the assumption and was thus used for the subsequent trend and ordinal regression analysis. Weighted results are reported to account for the PMA2020 sampling design and the variances of the covariates are adjusted accordingly. Graphs and analysis of the data were done using STATA statistical software SE version 14.1 (StataCorp. College Station, TX).

### Ethical considerations

PMA2020 survey activities received prior ethical approval certified through Ethiopia’s and John Hopkins University’s Institutional Review Board system and participants were consented for interviews. Approval for access of the anonymized data at the time of study conception was granted by Maastricht University under registration number FHML/GH_2019.093. As a secondary analysis using exclusively anonymized data, this study was determined not to qualify as human subjects research and waived from requiring approval for informed consent.

## Results

### Descriptive results and quality of counseling across the years

Table 1 summarizes the five survey rounds across the relevant independent variables and the classification of quality of counseling amongst current and recent contraception users. The most highly represented age group across all survey years was youth (20 to 24 years) [45.9% in 2018] followed by adults (25 to 49 years) [46.4% in 2018], while adolescents (15 to 19 years) were the least represented [7.7% in 2018]. Married females were the most highly represented demographic in all five survey rounds (91.2% married in 2018) and most of the women interviewed had given birth more than three times (49.9% in 2018). Rural dwellers comprised the majority respondents (73.4% in 2018), with the regions of Oromiya (32.6% in 2018) and Amhara (33.3% in 2018) with the highest share. Least represented were urban centers including Addis Ababa followed by the regions that comprise ‘other’ category at 5.4% and 3.1% respectively in 2018.

**Table 1:** Characteristics of female modern contraceptive users and quality of counseling by survey year, PMA2020 Ethiopia, 2014 to 2018.

	2014	2015	2016	2017	2018	Total	
	N (%)	N (%)	N (%)	N (%)	N (%)		
Total	1,834	2,289	2,348	2,223	2,354	11,048	
Age Category (years)							p-value*
Adolescents (15 to 19)	153 (8.4)	179 (7.8)	176 (7.47)	212 (9.6)	182 (7.7)	902 (8.2)	0.51
Youth (20 to 24)	902 (49.2)	1,111(48.5)	1,093 (46.6)	1,050 (47.2)	1,080 (45.9)	5,237 (47.4)	
Adult (25 to 49)	779 (42.5)	999 (43.7)	1,079 (46.0)	961 (43.2)	1,092 (46.4)	4,910 (44.4)	
**Education**							
None	854 (46.6)	1,047 (45.7)	1,048 (44.6)	933 (42.0)	925 (39.3)	4,807 (43.5)	0.27
Primary	670 (36.5)	833 (36.4)	863 (36.8)	846 (38.1)	929 (39.5)	4,141 (37.5)	
Secondary and higher	306 (16.7)	408 (17.8)	434 (18.8)	442 (19.9)	495 (21.0)	2,085 (18.9)	
**Wealth Quintile**							
Lowest	271 (14.8)	379 (16.6)	376 (16.0)	384 (17.3)	395 (16.8)	1,805 (16.3)	0.86
Low	283 (15.4)	401 (17.5)	421 (17.9)	403 (18.1)	431 (18.3)	1,938 (17.6)	
Medium	368 (20.1)	412 (18.0)	465 (19.8)	362 (16.3)	398 (16.9)	2,005 (18.1)	
High	409 (22.3)	494 (21.6)	482 (20.5)	527 (23.7)	525 (22.3)	2,437 (22.1)	
Highest	503 (27.4)	603 (26.3)	605 (25.8)	548 (24.7)	603 (25.6)	2,862 (25.9)	
**Marital Status**							
Married	1,689 (92.1)	2,067 (90.3)	2,096 (89.3)	2,013 (90.5)	2,146 (91.2)	10,012 (90.6)	0.23
Formerly in union	97 (5.3)	122 (5.3)	169 (7.2)	135 (6.1)	124 (5.3)	647 (5.9)	
Never married	48 (2.6)	100 (4.4)	82 (3.5)	76 (3.4)	84 (3.6)	390 (3.5)	
**Birth History**							
0 births	206 (11.3)	7 (13.9)	248 (10.6)	315 (14.2)	346 (14.7)	1,432 (13.0)	0.12
1 to 2 births	693 (37.8)	800 (35.0)	895 (38.1)	864 (38.9)	845 (35.9)	4,097 (37.1)	
3 plus births	935 (51.0)	1,171 (51.2)	1,205 (51.3)	1,044 (47.0)	1,163 (49.4)	5,518 (49.9)	
**Residence**							
Urban	483 (26.3)	604 (26.4)	606 (25.8)	573 (25.8)	625 (26.6)	2,891 (26.2)	0.95
Rural	1,350 (73.6)	1,685 (73.6)	1,742 (74.2)	1,651 (74.2)	1,729 (73.4)	8,158 (73.4)	
**Method Source**							
Public	1,542 (84.1)	1,886 (82.4)	1,990 (84.8)	1,789 (80.5)	1,837 (78.1)	9,045 (81.9)	0.05
Private	291 (15.9)	402 (17.6)	355 (15.1)	434 (19.5)	513 (21.8)	1,995 (18.1)	
**Method**							
Injectables	1,304 (71.1)	1,588 (69.4)	1,549 (66.0)	1,462 (66.0)	1,527 (65.0)	7,430 (67.3)	0.22
Implants	367 (20.0)	452 (20.0)	528 (23.0)	517 (23.2)	554 (23.6)	2,419 (22.0)	
IUD	34.2 (1.9)	52 (2.3)	46 (2.0)	68 (3.0)	53 (24.0)	254 (2.3)	
Pill	104 (5.7)	155 (6.8)	191 (8.1)	145 (6.5)	162 (6.9)	756 (6.9)	
Sterilization	15 (0.8)	25 (31,1)	15 (0.6)	13 (0.6)	26 (1.1)	95 (0.9)	
EC	10 (0.5)	17 (0.7)	19 (0.8)	17 (0.8)	31 (1.3)	94 (0.9)	
**Region**							
Addis Ababa	88 (4.8)	115 (5.0)	103 (4.4)	97 (4.4)	107 (4.5)	510 (4.6)	0.94
Amhara	591 (32.2)	68 (30.0)	758 (32.3)	742 (33.4)	783 (33.3)	3,561 (32.2)	
Oromiya	585 (31.9)	710 (31.0)	706 (30.1)	692 (31.1)	767 (32.6)	3,460 (31.3)	
SNNPR	394 (21.5)	574 (25.1)	584 (24.9)	476 (21.4)	496 (21.1)	2,523 (22.8)	
Tigray	128 (7.0)	130 (5.7)	128 (5.5)	118 (5.3)	128 (5.4)	632 (5.7)	
Other ^**¶**^	48 (2.6)	74 (3.2)	69 (2.9)	98 (4.4)	73 (3.1)	362 (3.3)	
**Quality of Counseling**							
No counseling	588 (32.1)	700 (30.6)	703 (29.9)	76 (34.5)	853 (36.2)	3,610 (32.7)	0.01
Poor counseling	450 (24.6)	478 (20.9)	564 (24.0)	521 (23.4)	540 (23.0)	2,553 (23.1)	
Intermediate counseling	247 (13.5)	193 (8.5)	282 (12.0)	207 (9.3)	268 (11.4)	1,197 (10.8)	
Good counseling	549 (29.9)	918 (40.1)	800 (34.1)	730 (32.8)	693 (29.4)	3,689 (33.4)	

To account for Performance Monitoring and Accountability 2020 (PMA2020) survey sampling strategy, weights are applied for representative results.

*Pearson chi square test.

^¶^ Other: Diri Dawa (urban center), Gambella, Afar, Benishangul-Gumuz, Somali. Abbreviations: EC: Emergency contraception, IUD: Intrauterine device.

Injectables were the most common method comprising 65.0% of method use in 2018 and the least common were emergency contraception and sterilization with 1.3% and 1.1% respectively in 2018. The public sector was the dominant source of contraceptives, supplying an estimated 80% of the methods being used.

There was a statistically significant increase in the proportion of females categorized under good quality counseling from 29.9% in 2014 to 40.1% in 2015 (Table 1, p = 0.01), only to see the percentage drop back down to 34.1% in 2016. Those rated in the intermediate categories of counseling quality proved to be the minority. The combined results for females within the no counseling and poor counseling reported comprise more than 50% of the recent or current modern contraception users, with no change nationwide across the survey years.

### Trends across age, marital status, birth history, education, and household wealth quintile

**Fig 2:**
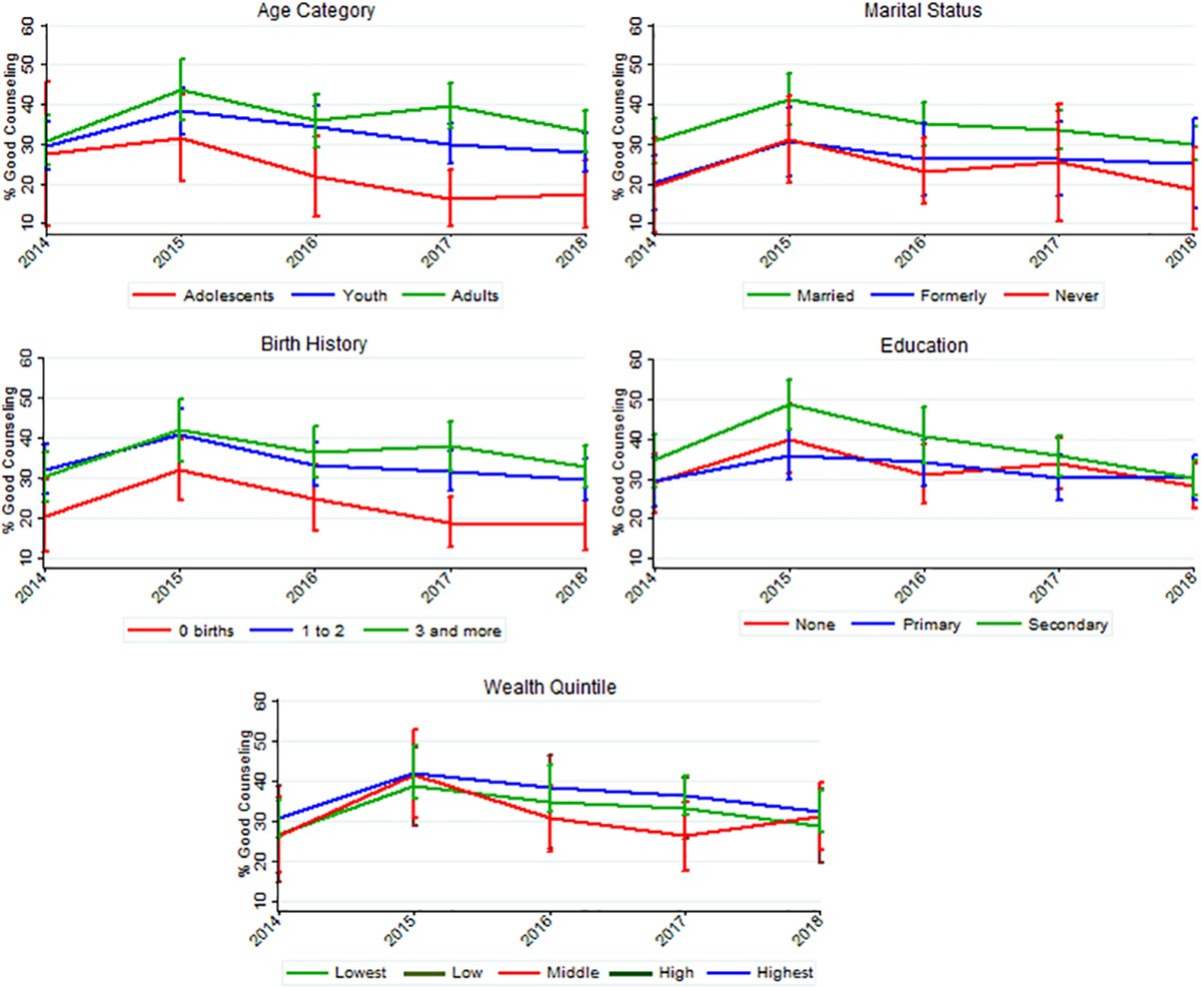
Trends of proportion of women receiving good counseling across key variables age, birth history, marital status, education, and wealth quintile. Data from Performance Monitoring and Accountability 2020 Ethiopia (2014 to 2018).

Fig 2 displays the proportion and 95% confidence intervals (CI) of women receiving good counseling across the rounds by each of the key variables. Across the age categories, there was no change over time, and before 2017, the difference in good counseling for adults, youth, and adolescents was not statistically significantly different as shown by the overlapping confidence intervals (Fig 2). As of 2017, the proportion of adolescents receiving good counseling was found to be significantly lower than for the youth and adult age categories. In 2018, only 12.3% (95% CI: 9.0, 25.3) of adolescents received good counseling, while 33.1% (95% CI: 27.9, 38.3) of adults were given counseling that was good with the criterion applied in this study.

Concerning the number of births, no significant changes in the proportion of women receiving good counseling were noted across the three categories. Between 2016 and 2018, women with zero births received inferior counseling when compared to women that have given birth more than once. In 2018, 32.7% (95% CI: 27.6, 37.9) of those with more than three births received good counseling while only 18.24% (95% CI: 12.6, 25.0) of those with no prior births were provided good counseling.

For education, there was a significant decrease in the proportion of women receiving good counseling amongst the secondary education level from 48.7% (95% CI: 43.2, 55.0) in 2015 to 30.3% (95% CI: 25.6, 34.9) in 2018 (Fig 2). There was also a significant decrease in women receiving proper counseling in the with primary education from 36.0% (95% CI: 29.7, 42.2) in 2015 to 30.2% (95% CI: 24.8, 35.7) in 2018. There was no significant change across the years in the group women with no education.

For each group within the marital status, there was also no change across time, yet in 2018 married women reported better counseling when compared to those that have never been married, 30.1% (95% CI: 25.9, 34.4) versus 18.7% (95% CI: 8.5, 30.0). Wealth quintile exhibited no change across any categories, nor were there significant differences between the quintiles.

### Trends across region and residence (urban/rural)

**Fig 3:**
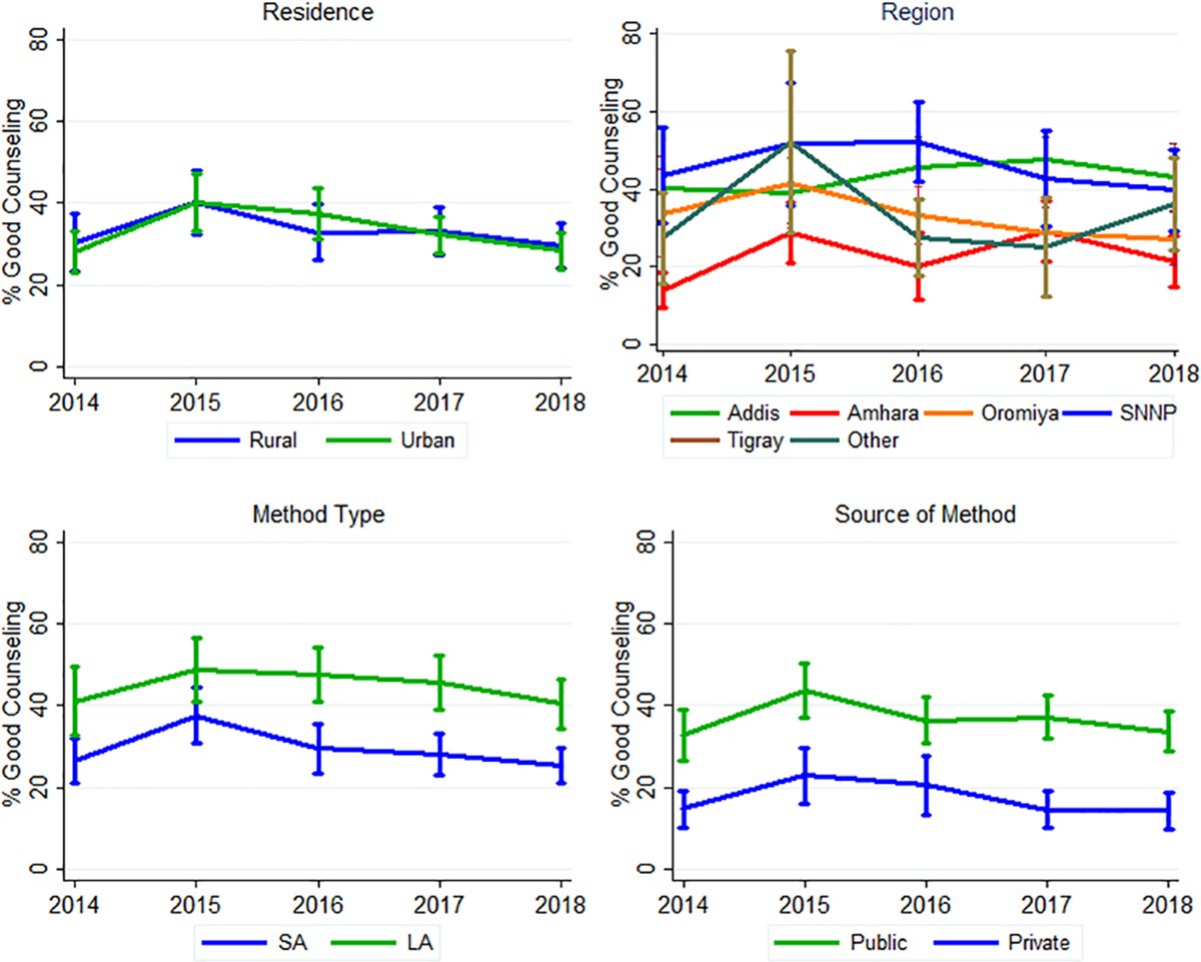
Trends of proportion of women receiving good counseling across region, residence, contraception method type, and source. Data from Performance Monitoring and Accountability 2020 Ethiopia (2014 to 2018).

None of the regions showed high numbers in the good counseling quality category (less than 50%), and no significant change was observed in the percentage receiving good counseling (Fig 3). The only region that reported significantly inferior counseling was Amhara (21.5% reporting good counseling, 95% CI: 14.8, 28.1) when compared to SNNPR (39.7% reported good counseling, 95% CI: 29.2, 50.2). Place of residence exhibited no change over time for urban and rural dwellers, nor was there a statistically significant difference between the two residences.

### Trends across contraception method type and source of contraception method

In terms of selected contraceptive methods, there was also no indication of change over time (Fig 3). There was a significant difference in the proportion receiving good counseling between users of the two methods, with long-acting method users having received superior counseling (in 2018: short-acting: 25.4% [95% CI: 21.1, 29.6] vs. long-acting 40.5% [95% CI: 34.5, 46.5]). With both private and public sector, there was no significant change over time. However, women who obtained contraception in the public sector received superior counseling versus women who reported getting their method from the private sector for their contraception needs (in 2018 private 14.3% [95% CI: 9.97, 18.7) versus in public sector 33.7% [95% CI: 28.9, 38.6].

### Factors associated with quality of family planning counseling: Ordinal regression results

Table 2 displays the descriptive results of the sample stratified by the predictor variables by quality of family planning counseling and the results of the ordinal regression analysis for the last survey year 2018. Results of the bivariate analysis showed that the correlates of age, birth history, residence, contraception source, and method type were all significant predictors of quality of counseling. When compared to youth, adolescents were less likely to receive good quality counseling (crude OR: 0.63, 95% CI: 0.43, 0.93, p = 0.019) while adults were 1.26 times more likely (95% CI: 1.03, 1.54, p = 0.024) to receive good quality. Those with no prior births were less likely to receive good counseling (crude OR: 0.49, 95% CI: 0.35, 0.70, p>0.001) when compared to the reference group of women with at least one past birth. Residents of Amhara (crude OR: 0.44, 95% CI: 0.27, 0.72, p<0.001) were significantly less likely to receive quality counseling when compared to SNNPR (reference category).

**Table 2:** Characteristics of female respondents by quality of counseling and ordinal logistic regression results, N = 2,354, Ethiopia, 2018.

	Quality of Counseling				Bivariate			Multivariable		
	Good (%)	Intermed. (%)	Poor (%)	None (%)	cOR	(95% CI)	p	aOR	(95% CI)	p
**Total (**n = 2,354)	69 (29.4)	268 (11.4)	540 (23.0)	853 (36.2)						
**Age (years)**										
Adolescent (15 to 19)	31 (17.3)	18 (9.7)	46 (25.7)	85 (47.2)	0.63	(0.43, 0.93)	0.02	0.91	(0.55, 1.50)	0.71
Adult (25 to 49)	361 (33.1)	133 (12.2)	258 (21.5)	3624 (33.2)	1.26	(1.03, 1.54)	0.02	1.12	(0.84, 1.47)	0.47
Youth (20 to 24)	300 (27.8)	116 (10.8)	259 (23.9)	405 (37.5)	Ref.			Ref.		
**Education**										
None	260 (28.1)	110 (11.9)	216 (23.3)	339 (36.6)	0.94	(0.74, 1.20)	0.64	0.70	(0.50, 0.97)	0.03
Primary	280 (30.2)	105 (11.4)	204 (22.0)	338 (36.4)	0.99	(0.78, 1.27)	0.96	0.80	(0.62, 1.03)	0.08
Secondary and higher	150 (30.3)	50 (10.1)	121 (24.4)	175 (35.3)	Ref.			Ref.		
**Wealth Quintile**										
Lower	120 (27.8)	51 (11.9)	93 (21.5)	168 (38.9)	Ref.			Ref.		
Lowest	114 (28.8)	61 (15.5)	77 (19.6)	143 (36.2)	1.12	(0.73, 1.73)	0.59	1.08	(0.72, 1.61)	0.72
Middle	125 (31.3)	52 (13.0)	86 (21.8)	135 (33.9)	1.21	(0.83, 1.76)	0.31	1.18	(0.84, 1.67)	0.34
High	140 (26.7)	52 (9.8)	147 (28.0)	186 (35.5)	1.02	(0.69, 1.50)	0.92	1.19	(0.78, 1.82)	0.42
Highest	194 (32.2)	52 (8.6)	137 (22.7)	220 (36.5)	1.12	(0.80, 1.59)	0.50	1.72	(1.10, 2.69)	0.02
**Marital Status**										
Never Married	16 (18.7)	4 (5.0)	16 (19.2)	48 (57.1)	0.54	(0.26, 1.14)	0.10	0.72	(0.36, 1.43)	0.34
Married, in union	646 (30.1)	247 (11.5)	500 (23.3)	753 (35.1)	1.26	(0.79, 2.02)	0.33	1.06	(0.71, 1.59)	0.78
Formerly Married	31 (25.1)	17 (13.6)	24 (19.6)	52 (41.8)	Ref.			Ref.		
**Birth History**										
0 births	63 (18.2)	33 (9.5)	65 (18.9)	185 (53.4)	0.49	(0.35,0.70)	<0.001	0.80	(0.52, 1.21)	0.28
3 and more births	381 (32.7)	148 (12.7)	252 (21.7)	383 (32.9)	1.13	(0.92, 1.39)	0.23	1.0	(0.76, 1.50)	0.73
1 to 2 births	249 (29.5)	87 (10.3)	223 (26.4)	285 (33.8)	Ref.			Ref.		
**Residence**										
Rural	515 (29.8)	215 (12.5)	389 (22.4)	611 (35.3)	1.16	(0.88, 1.52)	0.29	1.51	(1.04, 2.18)	0.03
Urban	178 (28.5)	52 (8.4)	153 (24.4)	242 (8.7)	Ref.			Ref.		
**Region**										
Addis Ababa	46 (43.2)	14 (13.1)	20 (18.8)	27 (24.9)	1.16	(0.68, 1.98)	0.58	1.28	(0.76, 2.18)	0.35
Amhara	168 (21.5)	74 (9.4)	197 (25.1)	345 (44.0)	0.44	(0.27, 0.72)	<0.001	0.51	(0.32, 0.81)	0.01
Oromiya	209 (27.3)	90 (11.7)	194 (25.3)	274 (35.7)	0.62	(0.38, 1.00)	0.05	0.67	(0.42, 1.08)	0.10
Tigray	46 (35.8)	12 (9.7)	36 (28.0)	34 (26.5)	0.89	(0.51, 1.53)	0.66	0.77	(0 .45, 1.30)	0.32
Other	27 (36.2)	5 (6.2)	13 (18.4)	29 (39.3)	0.68	(0.33, 1.38)	0.28	0.80	(0.40, 1.58)	0.50
SNNPR	197 (39.7)	73 (14.8)	80 (16.2)	145 (29.3)	Ref.			Ref.		
**Method Type±**										
Short-Acting	436 (25.4)	159 (9.3)	423 (24.6)	701 (40.8)	0.45	(0.36, 0.56)	<0.001	0.58	(0.46, 0.72)	<0.001
Long-Acting	257 (40.5)	109 (17.1)	117 (18.5)	151 (23.9)	Ref.			Ref.		
**Method Source**										
Private	73 (14.3)	33 (6.5)	96 (18.6)	311 (60.6)	0.28	(0.21, 0.38)	<0.001	0.31	(0.23, 0.41)	<0.001
Public	619 (33.7)	235 (12.8)	445 (24.2)	538 (29.3)	Ref.			Ref.		

Abbreviations: cOR: Crude odds ratio, aOR: Adjusted odds ratio, CI: confidence interval.

**±** Long-Acting: IUD, implants, sterilization, Short- Acting: injectables, EC, oral contraceptives.

The two most significant predictors in the bivariate analysis were contraception source and method. Those women reported getting their method from the private sector where 0.28 less likely (95% CI: 0.21, 0.38, p<0.001) to report good counseling when compared to the public sector.

In terms of contraception method type, those using short-acting methods were 0.45 less likely (95% CI: 0.36, 0.56, p<0.001) to receive good counseling when compared to women using long-acting methods. Furthermore, in the bivariate analysis, education, wealth quintile, marital status, and residence were not significant predictors.

In the multivariate analysis, age was no longer significant a predictor of quality counseling. On the contrary, education, wealth quintile, and residence became significant when adjusting for the other variables. Corroborating the findings of the bivariate analysis, marital status remained an insignificant predictor of quality of counseling in the adjusted analysis.

The likelihood of good quality counseling was the least among those who had no formal schooling as compared to those who had a secondary and above level of education (adjusted OR = 0.70, 95% CI: 0.50, 0.97, p = 0.03). Within the wealth quintile categories, the only significant finding was that women from the wealthiest quintile were 1.72 times more likely (95% CI: 1.10, 2.69, p = 0.017) to have received good quality counseling when compared to women living in the lower wealth quintile household. With residence, the change in significance revealed that women residing in rural areas were 1.51 times more likely in receiving good counseling when compared to those in urban areas (95% CI: 1.04, 2.18, p = 0.029). The change of significance in education, wealth quintile, and residence indicates that other variables were suppressing their significance.

Amhara remained significantly less likely to receive good counseling when compared to SNNPR (adjusted OR: 0.51, 95% CI: 0.32, 0.81, p = 0.005). Method source and contraception as with the bivariate analysis were also the two strongest predictors of increased quality of counseling in the multivariate model. Those women reporting acquiring their method from the private sector had worse counseling (adjusted OR: 0.31, 95% CI: 0.23, 0.41, p<0.001) when compared to the public sector. Those using short-acting methods were also more at risk of receiving lesser quality counseling when compared to users of long-acting methods (adjusted OR: 0.58, 95% CI: 0.46, 0.72, p<0.001).

## Discussion

This was the first study conducted in Ethiopia that used all five recent rounds of the PMA2020 female survey questionnaire to examine the quality of counseling from the perspective of female clients, an essential component of the accepted family planning quality framework posited by Judith Bruce [24]. The results revealed four key findings: First, more than half of Ethiopian women were not receiving enough information during family planning counseling when selecting their contraception method and no significant improvement over time was observed. This finding was independent of age, marital status, education, birth history, wealth quintile, residence, and region. Second, as expected, higher education and wealth quintile were associated with increased quality of family planning counseling. Third, women who resided in urban areas and Amhara were significantly less likely of receiving good quality when compared to rural dwellers and SNNPR. Fourth, women who obtained their contraception from the public sector and women who used long-acting contraceptive methods received significantly better-quality counseling from family planning practitioners.

Our findings on the trend analysis in the proportion of women receiving good counseling among contraceptive users corroborate Jain’s findings for the years 2005 to 2011 using DHS data [23]. Rwanda, a country which has experienced rapid increases in modern contraception use like Ethiopia, had higher MII scores with an increase during the two same years by almost 16 points while is decreased for Ethiopia by 2.8 points. This was attributed to the possibility that Rwanda might have focused its early family planning programs on both the provision of information to clients and increasing modern contraception use, while programs in Ethiopia seemed to have disproportionately emphasized the latter [23].

We identified several factors that influenced the odds of women receiving higher quality counseling. We did not confirm age as a factor influencing the odds of a woman receiving good quality counseling, which was surprising given that Ethiopian adolescents have been cited as being at higher risk of receiving poorer quality family planning services, including counseling [29]. Throughout Ethiopia, socially accepted sexual activity, childbearing, and contraception use are limited to the context of marriage, which was by far the highest represented category in marital status among our sample’s female respondents [30]. Therefore, this may explain why both marital status and birth history were not significant determinants of counseling quality amongst current and recent contraceptive users in this analysis. Even though age was not significant in the findings of this analysis, policies should continue to highlight the importance the reproductive and family planning needs of adolescent and youth since the country is mostly a young population.

We found evidence that women with higher education and those from wealthier households were more likely to receive good counseling when compared to women with no education and women with lower wealth, respectively. This is in line with findings in other countries [23]. In terms of education, Jain (2016) found that in the sub-Saharan Africa region, the MII scores increased with the education of the client. Regarding wealth, he found that increasing wealth was significantly associated with higher odds of receiving good counseling in Cameroon, Colombia, Egypt, Niger, and Nigeria [23]. Our finding of women’s educational status and its role in other positive family planning outcomes underscores the importance of promoting proper education amongst Ethiopian women.

MII analyses of female sterilization users in India, Colombia, and Honduras have indicated that women residing in urban areas had significantly higher odds of receiving good quality counseling than women living in rural areas [31, 32]. It is the reverse in Ethiopia, where we found that women residing in rural areas were most likely to receive good quality family planning counseling. In Ethiopia, about a third of the increase in modern contraception use over the past decade has been due to changes in contraceptive behavior in the rural population [33]. This can be explained by the rural-based health extension program in Ethiopia which has been in place since 2003 [34].

The bivariate analysis performed did not show the significant effect of education, wealth, and residence on the quality of counseling. In our sample, the public sector was used more by high educated women. Nevertheless, educated women have higher odds of getting good counseling in the public sector, as well, when compared to uneducated women. Thus, when we control for the source of method, the influence of education, wealth, and residence on quality becomes apparent.

In Ethiopia, the public sector plays a major role in the provision of family planning services and study on the technical quality of family planning counseling reported higher quality in public facilities when compared to the private sector [35, 20]. Our results indicated that women whose contraception source was the public sector had higher odds of receiving good counseling. This finding follows the pattern observed in other African countries such as Ghana and Rwanda, where the public sector also plays a dominant role in family planning services [23]. In the context of Ethiopia, the quality of the private sector should be of concern to healthcare regulators, policymakers, and family planning programmers since the private sector, including pharmacies and drug stores, have succeeded in providing access to short-acting methods and a preferred access points for underserved populations such as unmarried youth [36].

Our finding of injectables (short-acting method) as the predominant method of use (65% in 2018) is in line with findings in many Sub-Saharan countries including Ethiopia, where in 2016 injectables accounted for 64% of the method use [35]. Nevertheless, long-acting method users (IUDs, implant, sterilization) were identified as significantly more likely to receive good counseling. This finding may reflect the fact that long-acting methods such as implants and IUDs have been reported to be favored and promoted as the contraception option by family planning professionals [37]. Emergency contraception use in Ethiopia is not relevant to the general population and limited to the context of adolescent and young adult premarital sexual intercourse in mostly urban areas such as Addis Ababa [38]. Female sterilization is also a method that has not been the norm nor an important method of choice across the country [15]. Therefore, the categorization of all six modern contraception methods into two classifications is justified in this study, even when the context of obtaining EC or sterilization may be different from the other methods in their group.

This study revealed that the only significant regional variation was that Amhara residents were less likely to receive good counseling when compared to all other regions. In our sample, Amhara had the largest percentage of uneducated women when compared to the other regions, which may partly explain the results.

### Research and policy implications

Based on these findings, further research should focus on understanding the barriers and challenges in the provisioning of good counseling to women in Ethiopia. Qualitative studies focusing on the settings in which providers deliver counseling services to clients can shed light on counseling strategies and provide a more in-depth understanding of women’s experiences during family planning counseling. Such information cannot be captured solely in usual structured questionnaires of surveys.

Root causes of low quality in family planning services have been linked to gaps in provider training and skills, motivational factors such as low pay, and large volumes of clients [39]. In Ethiopia, a study in primary care centers in the Jimma zone identified a shortage of medical equipment, trained staff, and information education and communication materials in all of the family planning clinics, affecting service provision [40]. A study in a labor ward in Gondar, Ethiopia suggested that providers often want to provide quality care but lack the resources to do so. To improve quality, the study suggested that efforts to improve quality should focus on improving the working environment of providers in terms of infrastructure, addressing human resource shortages, remedying supervisory deficiencies, and skills training [41]. This may also relate to family planning counseling and should be investigated in further research.

Policies should encourage individuals that provide counseling across all sectors to receive comprehensive training not only with the technical skills, but the communication and interpersonal skills needed for effective counseling. The provision of teaching aid materials and appropriate spaces that foster privacy for counseling activities may support the provision of high-quality counseling across settings and demographics. Contraception providers and programmers can also take the initiative in adopting accepted guidelines, such as the World Health Organization’s ‘Quality of care in contraceptive information and services, based on human rights standards’ checklist, to support practices conducive to high-quality services [42].

### Limitations

There were important limitations to take into consideration. The MII, as the sole proxy of the quality of information exchange during family planning counseling, has its shortcomings. For example, the possibility exists that a woman’s response to the MII questions does not accurately capture the information exchanged during an earlier counseling session due to imperfect recollection. It may be that the capacity to recall and report information increases with a woman’s socioeconomic and educational status, which augments the significant positive impact of education and household wealth on counseling quality as seen in the results of the multivariate analysis. It is also critical to keep in mind that the three MII indicator questions do not capture all relevant topics related to FP counseling. Examples would be counseling on mixed-method use and sexually transmitted infection transmission prevention using male condoms. Monitoring information exchange by categorizing quality with the MII score from PMA2020 data alone does not provide information on the quality of counseling provided to high-risk groups such as commercial sex workers or Human Immune Virus (HIV) positive individuals whose contraceptive and counseling needs may be different from the general population.

Despite these limitations, measuring the different dimensions of quality in family planning is challenging, and the MII is a good starting point and an increasingly accepted method to monitor the information exchange that occurs during counseling from a client´s perspective.

## Conclusion

The results indicated that Ethiopia’s overall progress in modern contraceptive use has not been accompanied by a corresponding increase in the quality of family planning counseling. Room for improvement is evident as reflected by the finding of more than 50% of women receiving insufficient information across the basic information regarding side effects and availability of methods. Improving the quality of contraception counseling for women across all demographics, including wealth quintiles and education statuses, is a crucial strategy to support positive reproductive health outcomes with a rights-based focus. Therefore, the findings of this study are useful with its concentration on family planning service quality while focusing on proper information supplied to women during contraception counseling. Collaborative efforts by relevant stakeholders and policymakers across all sectors are necessary if the proportion receiving proper counseling in Ethiopia is to improve alongside other family planning indicators.

## Supporting information

S1 Table Distribution of recent and current modern contraceptive users in Ethiopia according to source of contraceptives and education, 2018. (DOCX)

S2 Table Distribution of recent and current modern contraceptive users in Ethiopia by region and education, 2018. (DOCX)

## Abbreviations

aOR: Adjusted Odds Ratio
cOR: Crude Odds Ratio
DHS: Demographic Health Survey
EC: Emergency Contraception
FP: Family Planning
FP2020: Family Planning 2020
HEW: Health Extension Workers
HIV: Human Immune Deficiency Virus
IUDs: Intrauterine Devices
LA: Long-acting
MII: Method Information Index
PMA2020: Performance Monitoring and Accountability 2020
SA: Short-acting
SNNPR: Southern Nations Nationalities and Peoples Region
