# Supplementary material for: Quality of Family Planning Counseling in Ethiopia: Trends and determinants of information received by female modern contraceptive users, evidence from national survey data, (2014- 2018)
Source: PLoS One. 2020 Feb 10;15(2):e0228714. doi: 10.1371/journal.pone.0228714 (PMC7010283; doi:10.1371/journal.pone.0228714)
Supplement: S1 Table — (DOCX) [file pone.0228714.s001.docx]

**Supplemental Table 1 -** Distribution of recent and current modern contraceptive users in Ethiopia according to source of contraceptives and education, 2018

|  | No education  N (%) | Primary  N (%) | Secondary/higher N (%) | Total  N (%) |
| --- | --- | --- | --- | --- |
| Public | 795 (86.0) | 733 (79.1) | 303 (61.5) | 1,832 (78.13) |
| Private | 129 (14.0) | 194 (20.9) | 189 (38.5) | 513 (21.9) |
| Total | 925 (100) | 927 (100) | 492 (100) | 2,354 (100) |
