# Supplementary material for: Quality of Family Planning Counseling in Ethiopia: Trends and determinants of information received by female modern contraceptive users, evidence from national survey data, (2014- 2018)
Source: PLoS One. 2020 Feb 10;15(2):e0228714. doi: 10.1371/journal.pone.0228714 (PMC7010283; doi:10.1371/journal.pone.0228714)
Supplement: S2 Table — (DOCX) [file pone.0228714.s002.docx]

**Supplemental Table 2 -** Distribution of recent and current modern contraceptive users in Ethiopia by region and education, 2018

|  | No education  N (%) | Primary  N (%) | Secondary/higher  N (%) | Missing  N (%) | Total |
| --- | --- | --- | --- | --- | --- |
| Amhara | 388 (41.9) | 261 (28.1) | 134 (27.1) | - | 783 (33.3) |
| Addis | 7 (0.8) | 29 (3.2) | 70 (14.2) | - | 107 (4.5) |
| Oromiya | 277 (29.9) | 347 (37.4) | 141 (28.6) | 2 (34.8) | 767 (32.6) |
| SNNPR | 197 (21.28) | 214 (23) | 86 (17.3) | - | 496 (21.1) |
| Tigray | 36 (3.91) | 44 (4.70) | 45 (9) | 4 (65.2) | 128 (5.4) |
| Other | 20 (2.2) | 34 (3.7) | 45(9.0) | - | 73 (3.1) |
| Total | 925 (100) | 929 (100) | 495 (100) |  | 2,354 |
